# Temperament and Character Personality Profile and Illness-Related Stress in Central Serous Chorioretinopathy

**DOI:** 10.1155/2014/631687

**Published:** 2014-02-16

**Authors:** Rupert Conrad, Franziska Geiser, Alexandra Kleiman, Berndt Zur, Andrea Karpawitz-Godt

**Affiliations:** ^1^Department of Psychosomatic Medicine and Psychotherapy, University of Bonn, Sigmund Freud Strasse 25, 53105 Bonn, Germany; ^2^Department of Clinical Chemistry and Clinical Pharmacology, University of Bonn, Sigmund Freud Strasse 25, 53105 Bonn, Germany

## Abstract

Psychological stress is a risk factor as well as a consequence of central serous chorioretinopathy (CSC). Impulsiveness, overachievement, emotional instability, and hard-driving competitiveness have been discussed as personality features in CSC patients. We investigated 57 consecutive CSC patients and 57 age- and gender-matched controls by means of the Symptom Checklist 90-R and the Temperament and Character Inventory. Somatic risk factors, illness characteristics, subjective assessment of severity of illness, and illness-related stress in different areas of life (work, private life) were evaluated. CSC patients showed significantly higher emotional distress as measured by the Global Severity Index. The CSC personality was characterized by lower scoring on the character dimension cooperativeness and the temperament dimension reward dependence. Cooperativeness as well as subjective assessment of severity of CSC has been recognized as significant predictors of illness-related work stress accounting for 30% of variance. Implicating competitiveness, hostility and emotional detachment, lower level of cooperativeness, and reward dependence support the existence of specific aspects of type A behaviour in CSC patients. Low perceived social support and loss of control may explain the significant contribution of this personality dimension to illness-related work stress. Treatment of CSC should thus incorporate psychoeducation about factors contributing to illness-related stress.

## 1. Introduction

Central serous chorioretinopathy (CSC) is an eye disease typically characterized by metamorphopsia, blurred vision, and micropsia with an annual age- and gender-adjusted incidence of about 5.8 cases per 100,000 people [1]. During the Second World War [2, 3] and shortly after the war [4, 5], a high incidence of one-sided retinopathy in the American military personnel was documented. Investigating physicians noticed a close association between combat-related stress and decrease of visual acuity [4, 5]. Several more recent studies reported an increased psychological distress in patients with CSC in comparison to healthy controls [6, 7]. On the one hand, stress coupled with sympathetic arousal has long been discussed as an important risk factor contributing to the development of the rare eye disease [8, 9]. On the other hand, stress can be seen as a consequence of the symptoms of CSC as sudden loss of visual acuity can cause a considerable degree of psychological distress [6, 7, 10]. Therefore stress may contribute to an unfavourable vicious circle in CSC patients counteracting therapeutic efforts. With regard to environment, a higher incidence of critical life events in CSC patients has been discussed as an important trigger [6, 11, 12].

Previous studies particularly underscore the role of personality in CSC patients in the development and maintenance of high work stress [6, 11, 13]. As far as personality is concerned, earlier studies indicated a tendency to overachievement, perfectionism, impatience, and hard-driving competitiveness described with a high degree of job involvement in CSC patients [11, 13], whereas more recent studies point to a personality characterized by emotional instability and insecurity [6, 7]. However, partly because personality was measured by inventories failing to satisfy accepted reliability and validity criteria, no coherent personality profile of CSC patients could be established yet [11, 13, 14].

In view of these facts we intended to investigate the characteristic personality profile underlying the development of CSC by means of an established psychometrically sound personality questionnaire the Temperament and Character Inventory (TCI) based on the psychobiological model of personality developed by Cloninger et al. [15, 16]. Temperament is thereby defined as the emotional core of personality, which is moderately heritable (i.e., the genetic, biological) and stable throughout life while character dimensions define the cognitive core of personality and refer to individual differences in self-concepts, goals, and values influenced by sociocultural learning and change throughout life [15, 16]. Thus, development of personality is mainly mirrored by change in character dimensions.

In regard to the previously described high degree of job involvement in CSC patients [11, 13], we assume CSC patients to be rather hard working, perseverant, and ambitious overachievers with a significantly higher degree of emotional distress. With regard to personality, CSC patients are expected to be curious, easily bored, impulsive, impatient [11, 13], more fearful, pessimistic, and emotionally unstable [6, 7]. Thus, we hypothesize CSC patients to score significantly higher than healthy control subjects on the novelty seeking, harm avoidance, and persistence as well as lower on the cooperativeness temperament dimension. Moreover, disease-related factors such as the extent of visual acuity loss, the subjective assessment of disease severity, and high persistence and low cooperativeness are expected to contribute significantly to illness-related work stress.

## 2. Methods

### 2.1. Sample Data

We consecutively enrolled all patients presenting with the diagnosis of CSC at the Department of Ophthalmology, Bonn University Hospital, within a period of 30 months. After a complete ophthalmological examination (Snellen visual acuity, funduscopy), the diagnosis was based on typical symptoms (visual decrease, metamorphopsia) and findings (serous retinal elevation) and was confirmed by fluorescence angiography (leaking point).

All patients were asked to complete questionnaires within 6 weeks after the initial onset of symptoms. Fifty-seven out of 78 patients (73.1%) completed the questionnaires. There were no significant differences in age, gender, or course of illness between participants and nonparticipants. In a previous publication we analyzed difficulties in emotional regulation in those 31 CSC patients of the sample, which were newly diagnosed and showed no (known) somatic risk-factors [12].

As a control group we used a group of 57 age- and gender- matched healthy volunteers. Only volunteers without current regular intake of medication, substance abuse or dependence, or psychiatric or organic illness were included. Matching took place on a concurrent basis, which means that each time a new patient was enrolled the best match was drawn from the pool of volunteers on the basis of gender and age. The colleague performing the matching procedure was blind to any hypothesis of our study as well as to the results of psychodiagnostic questionnaires. Study design was approved by the local ethics committee and all participants gave their informed consent.

### 2.2. Psychometric Instruments

General psychological stress was assessed by the German version [17] of the Symptom Checklist 90-R, which is a self-report instrument consisting of nine primary symptom dimensions (i.e., somatization, obsessive-compulsive disorder, interpersonal sensitivity, depression, anxiety, hostility, phobic anxiety, paranoid ideation, and psychoticism) with in sum 90 items, as well as a Global Severity Index indicating emotional distress. The Symptom Checklist 90-R has proven to be a reliable measure with adequate indices of concurrent criterion oriented and construct validity [17].

Additionally, patients' stress level due to symptoms of CSC in work and private areas of life during the last two weeks was measured on a five-point Likert scale (0 = no stress to 4 = maximum stress) by three items: (I) work, (II) family/partnership, and (III) leisure activities whereat the items (II) family/partnership, and (III) leisure activities were combined into one item named “private life” for further interpretation.

Personality was assessed using the Temperament and Character Inventory (TCI) [16] which distinguishes between four temperament (novelty seeking, harm avoidance, reward dependence, and persistence) and three character (self-directedness, cooperativeness, and self-transcendence dimensions).

### 2.3. Statistical Analysis

Statistical analysis was performed using the SPSS software package (SPSS v17.0, Chicago, Illinois, USA). Group comparisons concerning sociodemographic and illness-related data were performed using *t*-tests and Pearson's chi-squared tests (*χ* ^2^ tests) depending on the scale level.

The group effect on emotional distress and psychopathology as well as regarding personality dimensions was examined by analysis of variance (ANOVA). As the measurement of personality dimensions may be affected by anxiety or depression [16], we controlled for the influence of emotional distress including the Global Severity Index as a covariate in our ANOVA.

Bivariate Spearman's correlations were calculated to examine the relation between personality dimensions and disease characteristics with illness-related distress. Spearman's coefficients between 0.20 and 0.35 were evaluated as indicators of a weak association and coefficients between 0.36 and 0.55 as an association of medium strength. Furthermore, stepwise regression analysis was used to analyze predictors of illness-related work stress. *P* values <0.05 were considered statistically significant.

## 3. Results

The study sample consisted of 57 CSC patients (mean age: 46.8 ± 10.1 (SD) years) including 45 male and 12 female patients (sex ratio between male and female 4 : 1) and 57 gender- and age-matched control subjects (mean age: 43.4 ± 13.6 (SD) years). There were no significant differences between both groups concerning sample data (Table 1).

The mean time between the onset of symptoms and the time of examination was 5.5 ± 4.5 (mean ± SD) weeks (range 1–12 weeks). The patients' mean visual acuity (Snellen) was 0.53 ± 0.23 (calculated in decimal numbers; mean ± SD) and the mean severity of CSC on a visual analogue scale (1 = not severe to 4 = very severe) was 2.44 ± 0.80 (mean ± SD). Fifty-one patients (89%) presented with visual loss, 20 patients (35%) suffered from metamorphopsia, and 13 patients (23%) suffered from micropsia. With regard to risk factors, hypertension (21, 22) was found in 18 patients (32%), whereas all other risk factors such as sympathomimetic medication (23), corticosteroid medication (21, 22), antibiotic use (21), psychopharmacologic medication (22), pregnancy (21), allergic respiratory disease (22), organ transplantation (24), and excessive alcohol use (21) were found in less than 10% of the sample.

**Figure 1:**
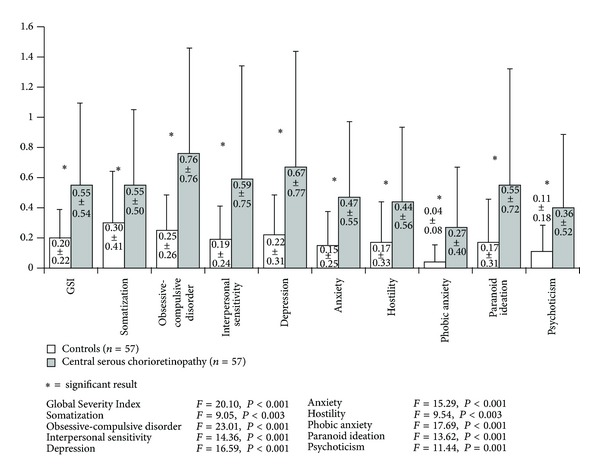
Showing heightened emotional distress and psychopathology, CSC patients scored significantly higher on all nine SCL-90-R symptom dimensions and the Global Severity Index compared to healthy controls.

Compared to healthy controls, CSC patients scored significantly higher on all nine SCL-90-R symptom dimensions representing emotional distress and psychopathology (Figure 1). Significantly higher distress as measured by the Global Severity Index (*F*(1,112) = 20.1, *P* < 0.001) could also be demonstrated.

**Figure 2:**
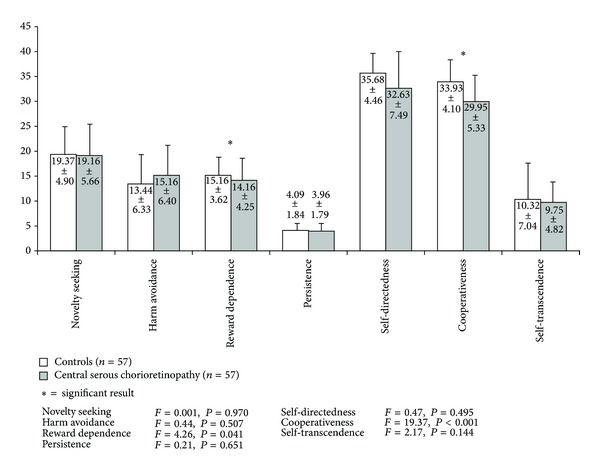
On the Temperament and Character Inventory (TCI) CSC patients scored significantly lower on the scales cooperativeness (*F*(1, 111) = 19.374, *P* < 0.001) and reward dependence (*F*(1, 111) = 4.263, *P* < 0.041) in contrast to controls.

CSC patients scored significantly lower on the scales cooperativeness (*F*(1, 111) = 19.374, *P* < 0.001) and reward dependence of the TCI (*F*(1, 111) = 4.263, *P* < 0.041) compared to controls (Figure 2). No significant between-group differences were presented on temperament scales novelty seeking, persistence, or harm avoidance.

Regarding the contribution of personality dimensions and disease characteristics to illness-related distress, CSC-related stress was assessed by the patients as highest at work (mean : 2.02 ± 1.19 (SD)). In private life it was perceived as less severe (mean: 1.10 ± 0.91 (SD)) (family/partnership: mean = 0.81 ± 1.0 (SD); leisure activities: mean = 1.39 ± 1.05 (SD)). Illness-related work stress was significantly correlated with harm avoidance (*r* = 0.289, *P* < 0.05), cooperativeness (*r* = −0.360, *P* < 0.01), and the subjective assessment of severity of illness (*r* = 0.447, *P* < 0.01), the latter being also in significant relation with illness-related stress in private life (*r* = 0.262, *P* < 0.05) (Table 2).

Finally, stepwise regression analysis revealed subjective assessment of severity of illness and cooperativeness being significant predictors of illness-related work stress, whereas visual acuity, reward dependence, and harm avoidance showed no significant influence (Table 3).

## 4. Discussion

The aim of the current study was to examine psychological distress in patients with CSC assuming that disease-related factors such as the extent of loss of visual acuity and the subjective assessment of severity of the eye disease as well as certain personality traits may contribute significantly to illness-related stress at work and in private life. The CSC underlying characteristic personality profile was established using the TCI developed by Cloninger et al. [15, 16].

Compared to healthy controls CSC patients showed a significantly higher degree of emotional distress, as we have shown in a smaller subsample of 31 CSC patients in a former study [12]. It is noteworthy that 20 patients (31.6%) showed a Global Severity Index (*t*-score) at least one standard deviation above the mean. CSC patients also scored significantly higher on all nine SCL-90-R symptom dimensions. This finding has also been reported in previous studies [6, 7, 10]. However, this measure does not assess specifically illness-related stress due to sudden loss of visual acuity, micropsia, and metamorphopsia.

With regard to the temperament and character personality profile, a striking difference in the character trait cooperativeness could be demonstrated revealing significantly lower cooperativeness in CSC patients. This TCI character dimension includes the subscales social acceptance, empathy, helpfulness, compassion, and pure-hearted conscience [16]. Individuals low in cooperativeness are prejudiced, hostile, critical, unhelpful, and opportunistic with the tendency to be inconsiderate of other people's rights or feelings. According to Friedman [18], hard-driving opportunistic competitiveness, aggression, and hostility triggered by minor frustrating events are the main facets of type A behaviour. Hence, our findings are in line with Yannuzzi [13] confirming type A like behaviour in CSC patients. Paal and Bereczkei [19] found a strong negative correlation between cooperativeness on the one hand and a person's tendency to deceive and manipulate other people for their personal gain on the other. The latter is subsumed in personality psychology under the term Machiavellianism. According to Cloninger [20], character traits are needed to integrate and control emotional drive as represented by temperament dimensions. A mature character is capable of solving a conflict between different urges and needs. Psychopathology develops if emotionality is not modulated successfully by a mature character [20]. From a clinical perspective, it is important to note that low cooperativeness is closely linked to unsatisfactory compliance with care and bad adherence to medication [16, 21].

Temperament is defined as heritable individual difference. In particular, reward dependence is viewed as heritable bias in associative learning in response to reward, namely, upholding of ongoing behaviours related to social attachment and dependence on approval of others [15]. As a result, reward dependent individuals have a heritable tendency to respond intensely to reward and learn to maintain rewarded behaviour. Low reward dependence, as shown in CSC patients, is associated with an antisocial personality, which is the reverse of the traits seen in passive-dependent personalities. Individuals on the low end of the reward dependence spectrum are socially and emotionally detached, content to be alone, independently self-willed, and usually rather practical and tough-minded and act for immediate gratification [15, 16]. Reward dependence has been confirmed to be determined in part by norepinephrine (NE) activity [22]. Cloninger suggests that increased NE levels are associated with low reward dependence. This hypothesis is supported by the finding that the locus coeruleus, which is a major source of central nervous system (CNS) noradrenergic output, is involved in the maintenance of behaviour by reward or nonpunishment [23]. The resulting low reward dependence goes well with our finding of low cooperativeness in CSC patients, since both traits are closely associated with the previously described type A behaviour observed in CSC patients in the past [13].

Interestingly, we could demonstrate a strong association between subjective assessment of stress due to CSC in the job and high harm avoidance, low cooperativeness, and high subjective severity of illness, the latter being also in significant relation with high illness-related stress in private life. While the temperament characteristic reward dependence is heritable, one might argue that lower cooperativeness is associated with lower social support, which might in particular cause problems in challenging situations in the working environment when impairment of vision may lead to the feeling of helplessness. By means of a regression analysis we aimed to get a deeper understanding of the specific contribution and relevance of personality traits, sociodemographic characteristics, and illness characteristics to the subjective work-related stress level in CSC patients. Altogether, the predictors accounted for 30.1% of variance. Interestingly, sociodemographic characteristics, sex, and age as well as illness characteristics such as illness duration and recurrence of illness did not contribute significantly to stress. However, our findings indicate that the subjective assessment of disease severity and the temperament dimension cooperativeness are significant predictors of illness-related work stress. Stress theory emphasizes that the subjective appraisal of a life event as unpredictable or uncontrollable is crucial to generate stress [24] and this may be even more so in a highly ambitious and competitive individual [18, 24]. Recent pathophysiological models of CSC [8, 9] suggest a cascade of events triggered by stress-induced hypercortisolism, which causes reduced choroidal flow, impaired hemorheology, and increased likelihood of platelet aggregation and microthrombus formation. This in turn leads to increased intraluminal pressure in the surrounding choriocapillaris, extravasation of serum, and tamponade of microvasculature finally resulting in neuroepithelial detachment.

CSC patients particularly at risk of the development of stress in the working environment might profit from psycho-education informing about the association of uncontrollability, helplessness, lack of supportive interpersonal communication, and stress and a careful explanation of mechanisms of disease, planned treatment and prognosis being the first step to enhance patients' self-efficacy. In patients with burnout symptoms, serotonin reuptake inhibitors may pose an additional pharmacotherapeutic option, which may also be advantageous with regard to cortisol-induced platelet aggregation [25].

## Figures and Tables

**Table 1:** Sociodemographic characteristics.

	Chorioretinopathy (*n* = 57)	Control group (*n* = 57)
Age		
Mean ± SD	46.8 ± 10.1	43.4 ± 13.6
Median	45.0	43.0
Gender		
Male	45 (78.9%)	45 (78.9%)
Female	12 (21.1%)	12 (21.1%)
Living situation		
Living alone	12 (21.1%)	18 (31.6%)
Living with a partner	45 (79.9%)	39 (68.4%)
Education		
No formal education	4 (7%)	1 (1.8%)
Secondary school	43 (75.4%)	39 (68.4%)
A levels/college	10 (17.5%)	17 (29.8%)
Working situation		
Other	3 (5.3%)	2 (3.5%)
Blue collar	29 (50.9%)	25 (43.9%)
White collar	24 (42.1%)	27 (47.4%)
Self-employed	1 (1.8%)	3 (5.3%)

**Table 2:** Spearman's correlations between personality dimensions, sociodemographic and illness characteristics, and illness-related stress in central serous chorioretinopathy patients.

	Illness-related stress	
	Work	Private life
Personality traits		
Novelty seeking	0.073	0.137
Harm avoidance	0.289*	0.127
Reward dependence	−0.035	0.022
Persistence	0.163	−0.155
Self-directedness	−0.240^(∗)^	−0.032
Cooperativeness	−0.360**	−0.002
Self-transcendence	0.129	−0.028
Sociodemographic/illness characteristics		
Sex	−0.116	−0.027
Age	−0.192	−0.179
Education^1^	−0.171	0.022
Employment^1^	0.022	−0.122
Partnership status^1^	0.135	0.141
Duration of illness	0.023	−0.071
Relapses (number)	−0.195	−0.083
Visual acuity (Snellen)	0.011	0.028
Severity of illness (subjective)	0.447**	0.262*

^(∗)^ *P* < 0.10; *P < 0.05; **P < 0.01.****

^1^Rank order as shown in Table 1:

education: 1: no education, 2: secondary school, 3: A levels; employment: 1: unemployed/other, 2: blue collar, 3: white collar, 4: self-employed; partnership: 1: single, 2: in a relationship.

**Table 3:** Significant predictors in stepwise regression analysis with dependent variable illness-related work stress (for the full list of sociodemographic/illness characteristics and personality dimensions as predictors see Table 2).

Predictors	*B*	SE B	*β*	*T*	*P*	*R* ^2^
Severity of illness (subj.)	0.629	0.165	0.425	3.803	<0.001	—
Cooperativeness	−0.085	0.025	−0.383	−3.427	0.001	adj. *R* ^2^ = 30.1*

*P < 0.001.
